# Sequence Variation in the E2-Binding Domain of HPV16 and Biological Function Evaluation in Tunisian Cervical Cancers

**DOI:** 10.1155/2014/639321

**Published:** 2014-06-17

**Authors:** Saloua Kahla, Lotfi Kochbati, Samia Hammami, Mohamed Badis Chanoufi, Mongi Maalej, Ridha Oueslati

**Affiliations:** ^1^Unit of Immuno-Microbio-Environmental and Carcinogenesis (IMEC), Faculty of Sciences, University of Carthage, Jarzouna, 7021 Bizerta, Tunisia; ^2^Radio-oncology Department, Salah Azaiz Institute, 1006 Tunis, Tunisia; ^3^Research Laboratory of Antimicrobial Resistance, Faculty of Medicine of Tunis, 1007 Tunis, Tunisia; ^4^Service of Gynaecology Obstetrics A, Center of Maternity and Neonatology, Hospital La Rabta, 1007 Tunis, Tunisia

## Abstract

HPV16 E2 variants have different effects on the transcriptional activity of the LCR. In this study, we examined the nucleotide and amino acid sequence variation within the HPV16 E2 gene and to correlate with disease progression. E2 gene disruption was detected by PCR amplification of the entire E2 gene using a single set of primers. Nucleotide variations were analyzed by bidirectional sequencing. mRNA expression patterns of E6 and E7 gene transcripts were evaluated by a reverse transcriptase-PCR method (RT-PCR). The detection of intact E2 genes was significantly higher among controls than cases (81.8% versus 37.5%, resp., *P* < 0.05). Among the E subgroup, variation at position 3684 C>A results in the amino acid substitution T310K and was more common among the E2 undisrupted cases (7/9; 77.7%), compared to controls (2/9; 22.2%). In addition, specific sequence variations identified in the E2 ORF at positions 3684 C>A were associated with increased viral oncogenes E6-E7 production. Besides HPV16 E2 disruption, the 3684 C>A variation within undisrupted E2 genes could be involved in an alternative mechanism for deregulating the expression of the HPV16 E6 and E7 oncogenes and appears to be a major factor contributing to the development of cervical cancer in Tunisian women.

## 1. Introduction

Development of cervical neoplasia is known to be causally associated with infection by high-risk types of the human papillomaviruses (hr-HPV). Infection with hr-HPV is a necessary but not sufficient cause of invasive cervical cancer, with additional virus-host interactions needed for cancer to develop [1–3]. The integration of a hr-HPV genome into the host chromosome is thought to be a key event in cervical carcinogenesis [4, 5] with integration often resulting in the loss of the viral E2 gene expression but with E6/E7 expression maintained or increased [6, 7] and overexpression of E6/E7 leading to immortalization and transformation of the host cell. The E2 protein is able to either activate or repress transcription of the E6 and E7 oncogenes by binding to the promoter region [8]. Three functional domains have been recognized in the E2 protein. The N-terminus contains the transactivation domain with amino acid residues (156–159), known to cooperate with the E1 protein in directing the synthesis of HPV16 DNA, linked to a C-terminal DNA-binding domain via a flexible hinge region [9]. The viral regulatory protein binds to multiple copies of the ACCN6GGT motif that occur in the long control region (LCR) of all HPVs [10, 11]. In genital HPVs, the E6-E7 promoter (such as P97 in HPV16) is suppressed by E2, because binding of the E2 protein can displace important cellular transcriptional activators (SP-1 and TFIID) from their adjacent binding sites [12, 13]. HPV16 with intact E2 in the episomal form are often found in cervical carcinomas [14, 15], reflecting the lack of suppression of the E6 and E7 genes caused by the E2 protein. Therefore, a factor that could contribute to differences in biological behavior of hr-HPV is DNA sequence variation. Taken together, such findings indicate that E2 gene disruption may not be a prerequisite for cervical carcinoma development. Among alternative mechanisms of enhanced viral oncogene expression are altered E2 functions resulting from variations in the E2 ORF, as reported in several studies [16–19]. E2 function can also potentially be altered by gene mutation/variation [17]. Data examining the E2 gene are however scarce. The E2 gene was chosen for detailed analysis because the products of this gene have significant roles in regulating the transcription of the viral oncoproteins E6 and E7. Theoretically, it is also possible that different HPV16 E2 variants have different effects on the transcriptional function of the LCR. In this study, we wanted to look at the nucleotide and amino acid sequence variation in the HPV16 E2 gene and to test the hypothesis that sequence variation is involved in disease progression. The transcriptional transactivation function of the isolated E2 variants and the specific genomic expression profiles was also examined.

## 2. Materials and Methods

### 2.1. Clinical Samples and Nucleic Acid Recuperation

Cervical cancer patients (*n* = 44) were recruited from the Radio-oncology Department of Salah Azaiez Institute (Tunis, Tunisia). Invasive cancer was staged according to criteria of the International Federation of Gynecology and Obstetrics (FIGO). The subjects ranged in age from 38 to 76 years (mean: 57.2 years). HPV16 DNA was present in 23 patients with squamous cell cancer and in one patient with adenocarcinoma. Benign cervical biopsies (*n* = 52) were obtained from consecutive women attending routine reproductive healthcare counseling in the Obstetrics and Gynecology Department of La Rabta Hospital (Tunis, Tunisia). The control group consisted of 29 women with a normal Pap smear and colposcopy as well as a negative HPV DNA test. The mean patient age was 41 years (range: 27 to 56 years of age). Biopsy specimens were suspended in 1 mL PBS (phosphate-buffered saline, pH 7.4) and then stored at −20°C until processed. DNA and RNA extraction were conducted using the QIAamp DNA Mini Kit and RNeasy Mini Kit (Qiagen, Hilden, Germany) following the manufacturer's instructions. The quality and quantity of the target nucleic acids were ascertained by agarose gel electrophoresis and spectrophotometry, respectively. DNA integrity was assessed by PCR amplification of the *β*-globin gene, which produces amplicons of 268 bp [20].

### 2.2. HPV16 Screening

The presence of HPV16 in cervical cells was detected by PCR using specific primers designed to amplify a 301 bp target in the L1 conserved region (synthesized by GENECUST, France). The PCR was conducted in a final reaction volume of 100 *μ*L reaction mixture containing 100 ng of DNA sample, 1.5 *μ*mol of each primer, 1.5 mmol Mg Cl2, 50 mmol KCl, 10 mmol Tris HCl, 200 *μ*mol of each dNTP (deoxynucleoside triphosphate), and 2.5 U of taq polymerase (Fermentas). PCR amplification was conducted for 40 cycles with denaturation at 94°C for 1 min, annealing at 58°C for 1 min, and extension at 72°C for 1 min. Amplification cycles were preceded by 5 min denaturation at 94°C and followed by 10 min final extension at 72°C. Each PCR experiment was performed with a negative control (water) and the appropriate positive controls for HPV16 (HPV16 Plasmids). The adequacy of the samples and the absence of PCR inhibitors were monitored by preliminary PCR amplifications with primers targeting the human *β*-globin gene [20]. All the samples gave adequate quality of genomic DNA. PCR products were examined by electrophoresis on 2% agarose gels stained with ethidium bromide and photographed under UV transillumination.

### 2.3. Physical Status of Viral Genome

Because integration of the HPV genome into the host DNA frequently disrupts the E2 gene, the physical state of the virus was investigated by PCR targeting the entire E2 gene as described by Bhattacharjee and Sengupta, 2006 [21]. Briefly, to distinguish the integrated viral DNA forms from the episomal forms, the integrity of HPV16 genomes was analyzed by PCR amplification with type specific primers targeting E2 ORF sequences in the region most frequently disrupted or deleted during the viral integration.

### 2.4. Reverse Transcription PCR for Detection of HPV16 E6 and E7 Transcripts

Samples were analyzed by RT-PCR according to the DNA results for each specimen. HPV16 positive samples were further subjected to amplification of the E6 and E7 transcripts [22]. RT-PCR was performed using primers for the constitutively expressed *β*-actin gene [22] as a positive control and subsequently for sample normalization. Details of the target genes, primer sequence, and amplicons sizes are shown in Table 1. The RT-PCR was performed in 50 *μ*L reactions using the adjusted amount of RNA template (1 *μ*g) with the one-step RT-PCR Kit (Qiagen). Reactions contained 0.6 *μ*M forward and reverse primers, 1x Qiagen one-step RT-PCR buffer, 400 *μ*M dNTP mix, and 2 *μ*L Qiagen one-step RT-PCR enzyme. The reaction was allowed to proceed for 30 min at 50°C. HPV16 type specific plasmids were used as positive controls. For each of the target genes, control reactions without template were performed in order to rule out contamination. Cycling protocols for all RT-PCR reactions are shown in Table 1. The amplicons were evaluated by 1.5% gel electrophoresis, marked by a 50 pb DNA ladder (Gene Ruler, Fermentas), stained with ethidium bromide, and visualized under UV light.

### 2.5. Semiquantitative RT-PCR Analysis

100 ng of cDNA from each sample was analyzed by semiquantitative RT-PCR analysis for quantitation of HPV16 E6/E7 oncogenes expression (cDNA levels reflect mRNA levels) by comparing the intensity and density of the ethidium bromide stained electrophoresis bands using the Doc-Print II software and Photo Capt software (VILBER LOURMAT, Marne-la-Vallée, France). Semiquantitative estimates of mRNA expression for each of the target genes were determined in relation to the expression of the *β*-actin gene in the same sample. Using the same amount of total cDNA for each sample allows for accurate comparison of the target genes. The significance of the expression levels of E6/E7 oncogenes with and without T310K variation was tested using Student's *t*-test.

### 2.6. HPV16 E2 Gene Sequence Analysis

In order to evaluate the HPV16 integrity and nucleotide sequence alterations within the intact E2 gene, we performed bidirectional sequencing of the PCR products obtained using the specific primers [21]. Briefly, PCR products were purified using the Spinklean PCR Purification kit according to the manufacturer's instructions (Biomatik). Ten *μ*L of the purified PCR products was sequenced with forward and reverse primers targeting the E2 ORF region using the BigDye Terminator Cycle Sequencing Kit (Applied Biosystems). The products were analyzed with ABI PRISM 310 DNA Sequencer (Applied Biosystems, Foster City, CA). The resulting sequences were compared with sequences in the NCBI database (http://www.ncbi.nlm.nih.gov/) using the BLAST program from the same website to determine if they contain human DNA sequences.

Multiple sequence alignment of the E2 sequences and the reference HPV16 sequence (HPV16 R) from the HPV16 Sequence Database (Los Alamos National Laboratory) was done using the multiple sequence alignment program Bio Edit in order to detect nucleic acid variations.

### 2.7. Statistics

The relationships between the different variables were assessed using Fisher's Exact Test. The data were analyzed statistically using Student's *t*-test. Values of *P* < 0.05 were considered to indicate statistical significance. The analyses were carried out using SPSS, version 18.0 for windows.

## 3. Results

### 3.1. HPV16 Typing

**Figure 1:**
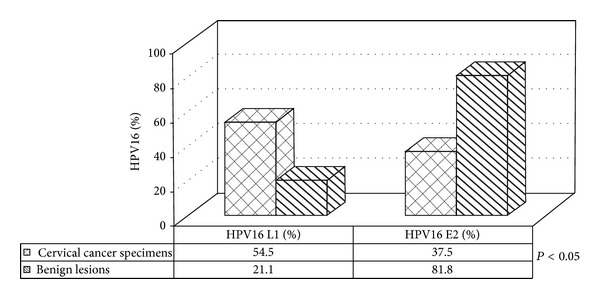
Presence of HPV16 L1 and E2 genes in cervical carcinomas and controls. *P*: Fisher's Exact Test.

Using the specific primers targeting the late L1 conserved region, HPV16 DNA was identified in 35 of the 96 cervical biopsies examined. HPV16 DNA was identified by PCR and typing in 24/44 (54.5%) cervical cancer biopsies and in 11/52 (21.1%) benign biopsies (Figure 1).

### 3.2. Distribution of HPV16 E2 Episomal Forms in Cervical Specimens

All cervical specimens could be amplified with the beta globin primers. In cervical carcinomas amplification was observed in 9/24 (37.5) samples using the E2 ORF primers and in 9/11 (81.8%) of benign samples. Failure of E2 amplification despite amplification of an internal control with a larger product size is consistent with disruption of the E2 gene.

Detection of the intact E2 gene was significantly more frequent in benign lesions than in cervical carcinomas (*P* < 0.05, Fisher's Exact test) (Figure 1).

### 3.3. Identification and Analysis of HPV16 E2 Sequence Variation

The entire E2 gene was sequenced in all samples. The numbering of the nucleotide variations was based on the reference sequence of HPV16 (HPV16 R), which is available in the HPV16 Sequence Database (Los Alamos National Laboratory). The results of sequence analysis of the E2 gene region from 9 cases and 9 controls are summarized in Table 2. Of the 9 cases, 5 (55.5%) had the HPV European (E) variant and 4 (44.4%) had the Africain-2 variant, while 8 (88.8%) of the controls also had the E variant and 1 (11.1%) had the Africain-2 variant. The 25 mutations were distributed throughout the entire E2 gene and 17 resulted in amino acid changes.

A total of 12 DNA sequence variations were identified in the E2 gene region encoding the amino terminal domain and 4 variations were detected in the hinge domain. In addition, 9 DNA sequence variations were found in the transactivation domain including one new sequence change at position 3790 (Table 2). This analysis shows that the amino acid sequence at or near to the E2 hinge region is more frequently conserved. We also correlated the clinicopathologic characteristics with HPV16 E2-variant category to evaluate the possible association of the HPV16 E2 variants with clinical behavior in cervical cancers. There was no significant difference in the distribution of HPV16 E2 variants according to cervical specimen groups. Similarly, none of the clinical parameters showed a significant difference associated with HPV16 E2-variant status including FIGO stage and histologic cell types (Table 3). However, we did show a significant trend between age groups and HPV16 E2 variants (*P* = 0.02).

### 3.4. Correlation between E2 DNA-Binding Domain Variation and E6/E7 Oncogene Expression

The expression of E6/E7 viral oncogenes was focused in every sample according to the mutation observed but we did not reach a significant level except for T310K mutation. Add to this that the frequency of the other mutation from benign to malignant specimens raises the possibility that variants with those alterations could be involved in the development of high grade intraepithelial and invasive disease from benign lesions. Since no case was identified for the mutations R165Q and N344E in the benign lesions, no conclusion can be made concerning that these mutations can be implicated in the malignant progression.

**Figure 2:**
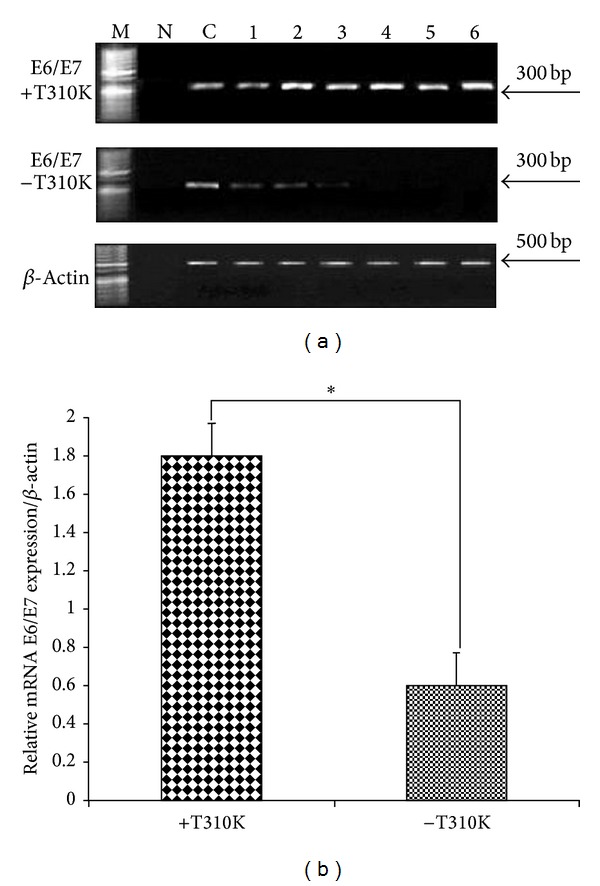
(a) RT-PCR products of HPV16 E6/E7 in episomal forms from cervical positive samples with and without T310K variation. Lanes 1–6 are shown as examples (amplicon length 300 bp). M: size markers of 50 bp ladder; N: negative control (no template); C: positive control (HPV16 plasmid). (b) Specific gene expression and quantification of HPV16 E6/E7 relative to *β*-actin expression levels in episomal forms with and without T310K variation. Bars represent mean ± SEM. *Significantly elevated expression (*P* < 0.05), Student's *t*-test.

In the DNA-binding domain of E2, sequence variation at position 3684 results in the amino acid substitution T310K that was detected in 7 of 9 cervical cancer samples (77.7%), compared with only 2 of 9 controls (22.2%) (Table 2). The association of T310K variants with high expression of E6/E7 oncogenes suggests that E2 variation may be an alternative mechanism for deregulation of viral oncogenes expression (Figure 2). These data strongly suggest that the T310K E2 mutant may reverse its regulation activity on viral oncogenes expression and may be biologically relevant in vivo.

## 4. Discussion

Certain types of HPV are considered as high-risk due to their strong association with cervical carcinogenesis and their ability to integrate into the host genome [23]. Previous studies of HPV integration into the host genome have focused mainly on HPV16, the type that confers the highest risk of cervical cancer and also the type most commonly detected in women with normal cervical cytology [24]. Identifying the presence of intact E2 genes in almost 37.5% of HPV16 positive cervical cancer cases has paved the way for new paradigms of cervical carcinogenesis. However, E2 disruption is not a prerequisite for the development of invasive disease as intact E2 genes have been identified in invasive lesions [25, 26]. Recently, independent studies have provided evidence that specific intratype HPV genome variation, especially in HPV16, may influence the persistence of infection and progression to cancer [27]. Types and variants are defined as those strains with at least 90% similarity and generally greater than 95% similarity, respectively [28]. Four phylogenetic branches of HPV16 have been identified: European (including an Asian clade), Asian/American, African-1, and African-2, named according to their geographic prevalence [29]. These variants are geographically distributed and suggest that HPV has coevolved with human population migration [30, 31]. To date, little HPV variant research has been performed in Tunisia or North Africa. Tunisia presents an interesting model to study HPV variants due to the history of population movements through North Africa, as well as North Africa's striking intra- and intercountry incidence differences. One would expect higher rates of European HPV16 variants in Tunisia due to its history with Europe and this was confirmed in our study, as non-European HPV16 variants have a stronger association with more aggressive cancers that are diagnosed at later stages [32]. It should also be noted that cases infected with non-European variants of HPV16 had a slightly younger age at diagnosis. The non-European variants of HPV16 may pose a 2- to 9-fold increased risk of HSIL and cervical cancer, depending on the respective populations [33]. Therefore, identification of HPV16 variants may be important for the design of newer diagnostic and therapeutic interventions in cervical cancer as well as for vaccine development strategies. Additionally, any change in the sequences of E2 gene may lead to altered biological function of their proteins, which in turn may influence the natural history of the infection. Among such cases, several variations were noted in the E2 sequences, chiefly within the region encoding the DNA-binding domain. Multiple sequence alignment analysis revealed that specific nucleotide variations were associated with non-European variants such as African-2 variants. The E2 protein is a strong transcriptional activator and greatly increases viral DNA replication by colocalizing the viral E1 protein to the origin of replication. The region of the E2 protein required for this association with the E1 protein is located near the N-terminal transactivation domain [34, 35]. The absence of variations in this region (amino acid positions 18–41), except for one variation at 35 amino acid position (His>Gln), in our study suggests that the binding capacity of E2 to E1 could remain unaltered, also suggesting that the replication efficiency of E2 would remain unaffected. The functional importance of mutations within the hinge region is less clear, although they could potentially alter the three-dimensional relation between transactivation and DNA-binding domains. This possibility is of particular relevance to the P219S (3410 C to T) mutation, which occurred at approximately the same frequency in cervical cancer specimens and benign lesions, because the Pro>Ser replacement at this position could significantly alter the secondary and tertiary protein structures. The frequency of this mutation raises the possibility that variants with this alteration could be involved in the development of high grade intraepithelial and invasive disease.

As a 3684 C>A (T310K) within the DNA-binding domain of E2 was commonly associated with cervical cancer in our study (7 of 9 cases compared to 2 of 9 controls), genetic instability of the E2 gene may enhance overexpression of E6/E7 oncoproteins resulting in rapid progression to aggressive malignancy. This was in line with Giannoudis et al. [17] but contradictory to Bhattacharjee and Sengupta [21]. It has been suggested earlier [36, 37] that high concentrations of episomal E2 could repress the expression of the viral oncogenes by preventing the binding of cellular transcription activators to their respective sites within the P97 promoter. As shown previously, we reported a direct interplay between the HPV16 E2 and E6/E7 proteins. We consistently observed that E2 protein has a repressive effect in vivo on the expression of the E6/E7 oncogenes [26]. Thus, it has been suggested that the loss of E2 function as a consequence of disruption or mutation (as in this study) could result in the upregulation of the viral promoter with increased expression of the oncogene transcripts [38]. The effect of this mutation on the biological function of E2 seems to be very critical, since the other amino acid exchanges located in the transactivation domain and the hinge regions of E2 have little impact. Overall, our study, like that of Giannoudis et al. [17], supports contentions that sequence variations in the HPV16 E2 region may be a principal factor involved in the enhanced expression of the E6 and E7 oncoproteins. One possible mechanism is that the interaction between cellular transcription factors, the E2 protein, and the viral promoter/enhancer region may be altered by the T310K variant. This hypothesis would fit well with our data showing high expression of episomal forms of the E6/E7 genes. Although, some studies have noted that this variation C3684A could be related to the conformational alterations of DNA structure. This variation lies adjacent to the DNA-binding helix of the E2 protein and therefore could alter the conformational structure of this helix as well as the conformation of the E2 protein. Therefore, the T310K mutation, prominent in cervical cancer variants, might affect the three-dimensional structure of this helix and hence its ability to bind to DNA. The E2 protein exists in solution and binds to the target DNA as a dimmer, whereas the C-DNA-binding domain consists of a dimeric *β*-barrel structure with a pair of symmetrically disposed *α*-helices that bind and bend the DNA [39]. However, in *β*-barrel structures the hydrophobic residues are oriented into the interior of the barrel to form a hydrophobic core and the stability of the *β*-barrel depends largely on the interaction of the inner hydrophobic amino acid residues. The mutation from Thr to Lys at aa 310 can decrease the hydrophobic property and subsequently destabilize the dimeric structure of E2, which is possibly responsible for the lessening of DNA-binding activities. The T310K could be functionally important given that the DNA-binding activity of E2 is important for its function. In support of our findings, another study also identified that the mutation from Ala>Val at amino acid position 338 of HPV2 E2, which would change the hydrophobicity and/or tertiary structure of E2, will lead to a modification of its interaction with the chromatin and thus modulate its transcriptional regulation activity. Although the point mutations in TAD and in the hinge region within this E2 mutant do not affect DNA-binding and transcriptional regulation, their influence on viral genome replication cannot be excluded [16]. However, the sequence variations in the E2 gene may not be the major mechanism responsible for enhancing the expression of E6 and E7 oncoproteins [40]. Some studies have also observed elevated levels of E6 and E7 in primary or metastasizing tumors carrying intact E2, due to deletions or point mutations affecting one or more binding sites of the transcription factor YY1 in the viral LCR [41, 42]. Our findings suggest that disruption of the E2 gene is not necessary for deregulation of the expression of the E6/E7 viral oncogenes and that the E2 variants may be an alternative mechanism for deregulating the expression of viral oncogenes. In this study, we have provided evidence that the mutation at T310K in HPV16 E2 decreases E2 DNA-binding affinity and reverses its transcriptional regulation activity on the viral early promoter.

## 5. Conclusions

In summary, the results presented here suggest that the T310K variant may be linked to high expression of the viral oncogenes and progression to cervical cancer and can be assessed in high-throughput manner facilitating the discovery of markers that predict cervical progression. Such a study would provide valuable information, as the level of the HPV16 E6-E7 transcripts and the detection of E2 mutations, with DNA physical state detection, could serve as an additional mechanism for evaluating risk for the development of cervical carcinoma.

## Figures and Tables

**Table 1:** Polymerase chain reaction and reverse transcription primers, product length, and programs.

	Designation	Product length (bp)	PCR program
Primers for PCR assay			
*β*-Globin sense	5′-CAACTTCATCCACGTTCACC-3′	268	95°C 1′, 55°C 1′, 72°C 1′; X 40
*β*-Globin antisense	5′-GAAGAGCCAAGGACAGGTAC-3′		
HPV16 L1 sense	5′-GCAAGCAACAGTTACTGCGACGT-3′	301	94°C 1′, 58°C 1′, 72°C 1′; X 40
HPV16 L1 antisense	5′-GCAACAAGACATACATCGACCGG-3′		
HPV16 E2 sense	5′-ATGAAAATGATAGTACAGAC-3′	1026	95°C 1′, 50°C 2′, 72°C 1′ 30 s; X 35
HPV16 E2 antisense	5′-CCAGTAGACACTGTAATAG-3′		
Primers for RT-PCR assay			
*β*-Actin sense	5′-AGCCATGTACGTTGCTATCC-3′	500	94°C 30 s, 50°C 30 s, 72°C 1′; X 30
*β*-Actin antisense	5′-TTGGCGTACAGGTCTTTGC-3′		
HPV16 E6 sense	5′-TTACCACAGTTATGCACAGA-3′	300	94°C 30 s, 50°C 30 s, 72°C 1 min; X 30
HPV16 E6 antisense	5′-ACAGTGGCTTTTGACAGTTA-3′		
HPV16 E7 sense	5′-AGAAACCCAGCTGTAATCAT-3′	300	94°C 30 s, 50°C 30 s, 72°C 1 min; X 30
HPV16 E7 antisense	5′-TTATGGTTTCTGAGAACAGA-3′		

**Table 2:** Sequence variations and amino acid substitutions of HPV16 E2 variants compared to the reference sequence.

Polymorphism					Malignant (*n* = 9)	Benign (*n* = 9)
Domain	Nucleotide		Amino acid residue			
	Number	Change	Number	Change		
Amino terminal (Transactivation domain)	2860	C>A	35	His>Gln	4	1
	2926	A>G	57	Gln∗	6	4
	2938	A>G	61	Thr∗	5	3
	3043	C>T	96	Asp∗	4	1
	3159	C>A	135	Thr>Lys	2	0
	3161	C>T	136	His>Tyr	3	1
	3182	G>A	143	Ala>Thr	2	0
	3249	G>A	165	Arg>Gln	4	0
	3362	A>G	203	Asn>Asp	2	0
	3377	C>G	208	Pro>Ala	1	0
	3384	T>C	210	Ile>Thr	5	8
	3410	C>T	219	Pro>Ser	7	6

Hinge	3431	G>A	226	Ala>Thr	4	1
	3449	G>A	232	Glu>Lys	3	1
	3516	C>A	254	Thr∗	2	1
	3517	T>C	254	Thr>Asn	4	1

Carboxy terminal (DNA-Binding Domain)	3538	A>C	261	Ser∗	4	1
	3566	T>G	271	Phe>Val	2	0
	3684	C>A	310	Thr>Lys	7	2
	3694	T>A	313	Thr∗	2	0
	3706	T>C	317	Ser∗	4	1
	3778	G>T	341	Trp>Cys	3	0
	3787	C>A	344	Asp>Glu	4	0
	3790	A>T	347	Ile>Phe	3	1
	3805	T>G	350	Val∗	2	0

*No amino acid change.

**Table 3:** Clinicopathologic features of cervical cancer cases.

Physical status of HPV16 DNA				
Trait	Category	European (%) *N* = 13	Non-European (%) *N* = 5	*P value
Patients	Cervical carcinoma	5	4	0.2
	Benign	8	1	

Age group (years)	≤64 years	2	4	0.02
	>64 years	11	1	

Stage FIGO	Early (I/II)	5	3	0.4
	Late (III)	0	1	

Cell type	Squamous cell carcinoma	5	4	—
	Adenocarcinoma	0	0	

*P: two-sided Fisher's Exact Test.
